# Stabilization of Submicron Calcium Oxalate Suspension by Chondroitin Sulfate C May Be an Efficient Protection from Stone Formation

**DOI:** 10.1155/2013/360142

**Published:** 2013-12-08

**Authors:** Jun-Jun Li, Jun-Fa Xue, Jian-Ming Ouyang

**Affiliations:** ^1^College of Pharmacy, Guangdong Pharmaceutical University, Guangzhou 510006, China; ^2^Institute of Biomineralization and Lithiasis Research, Jinan University, Guangzhou 510632, China

## Abstract

The influences of chondroitin sulfate C (C_6_S) on size, aggregation, sedimentation, and Zeta potential of sub-micron calcium oxalate monohydrate (COM) and calcium oxalate dihydrate (COD) crystallites with mean sizes of about 330 nm were investigated using an X-ray diffractometer, nanoparticle size Zeta potential analyzer, ultraviolet spectrophotometer, and scanning electron microscope, after which the results were compared with those of micron-grade crystals. C_6_S inhibited the conversion of COD to COM and the aggregation of COM and COD crystallitesis; it also decreased their sedimentation rate, thus increasing their stability in aqueous solution. The smaller the size of the COD crystallites, the easier they can be converted to COM. The stability of sub-micron COD was worse than that of micron-grade crystals. C_6_S can inhibit the formation of calcium oxalate stones.

## 1. Introduction

The formation of urinary stones is closely related to supersaturation, nucleation, growth, and aggregation of stone salt. Compared with the urine of stone patients, normal urine has more types of inhibitors with higher concentration and stronger activity. These inhibitors include some small-molecule inorganic salts such as citrate and pyrophosphate and urinary macromolecules such as glycosaminoglycan (GAG), nephrocalcin, Tamm-Horsfall protein, and prothrombin fragment 1 [1–5]. As the main component of urinary stones, calcium oxalate (CaOxa) mainly exists in the form of calcium oxalate monohydrate (COM) and calcium oxalate dehydrate (COD). In the urine of patients afflicted with urinary stones, the existing probability of COM crystallites is much higher than that in healthy controls [6].

A study showed that CaOxa crystals in urine only take 3 min to 4 min to flow through the nephron [7] and about 12 min to pass through the pelvis. Within such a short time, the crystal could not grow into a pathological size (larger than tens of microns). Rapid aggregation of the crystals is an important factor in CaOxa stone formation [8, 9].

GAG is an important urinary macromolecule that inhibits urinary stone formation [2]. Urinary GAGs originate from two sources. The first source of urinary GAGs is the serum, which is filtered through the kidney into the urine. The electrophoretic types of GAGs in urine are similar to those present in the serum; moreover, the excretion of urinary GAGs increases along with the increment of GAG concentration in the serum [10–12]. The GAGs in the serum originate from degradation products of proteoglycans in connective tissues, such as the cartilage, intervertebral disk, cornea, skin, blood vessels, hemocyte, and thrombocyte, as well as other organs, such as the brain, kidneys, and the liver. The GAGs in these tissues and organs often combine with proteins and exist as proteoglycans. The second source of urinary GAGs is the urinary tract. The urinary tract, especially the bladder surface, can secrete GAGs [13]. Consequently, the concentration of urinary GAG gradually increases down the urinary tract. The GAGs formed in the urinary tract can form a GAG layer on the surface, inhibiting the adhesion of urinary crystallites to cells and the formation of renal calculi.

GAGs excreted in the urine include eight components: chondroitin sulfate A (C_4_S), chondroitin sulfate C (C_6_S), chondroitin (CH), hyaluronic acid (HA), heparan sulfate (HS), dermatan sulfate (DS), heparin (HP), and keratin sulfate (KS) [14–16].

The concentration of GAGs in the 24 h urine of patients afflicted with urinary stones is significantly lower than that in controls. The former exhibits a concentration of 2.97 ± 0.43 mg/L (male) and 2.32 ± 0.24 mg/L (female), whereas the latter shows a concentration of 8.22 ± 0.60 mg/L (male) and 7.97 ± 0.43 mg/L (female) [17]. The concentrations of GAGs (5.62 *μ*g/mg) and chondroitin sulfate (2.81 *μ*g/mg) in the urine of the control subjects were higher than those (4.75 and 1.67 *μ*g/mg, resp.) of the stone-forming patients [16]. In the literature [18], the concentration of GAGs in the control subjects' urine (6.20 ± 0.68 mg/dL) was higher than that in the stone-forming patients (uric acid stones: 3.77 ± 0.68; CaOxa stones: 5.16 ± 0.55; CaP stones: 3.88 ± 0.79 mg/dL). The incidence of pediatric urolithiasis is less in adults due to the higher GAG concentration in children's urine (children: 10.2 ± 0.58 mg/L, adult: 5.06 ± 0.47 mg/L) [19].

GAGs can inhibit the nucleation, growth, and aggregation of CaOxa stones [20]. Out of the eight components of GAGs, chondroitin sulfate (CS, including C_6_S and C_4_S) is responsible for the main inhibitory effect of GAGs [14]. About 55% of GAGs in the urine of the control subjects were CS, whereas only 35% of those in the urine of stone-forming patients were CS [16]. C_6_S is a linear polysaccharide polyanion. Each repeating disaccharide unit has a negatively charged sulfate group and carboxylic group [21].

Only a few reports on submicron CaOxa crystals exist [22, 23]. Therefore, the effect of C_6_S on aggregation and sedimentation of COM and COD crystallites with mean size of about 330 nm was investigated in this article to study further the formation mechanism of urinary stones.

## 2. Experimental Section

### 2.1. Reagents and Apparatus

C_6_S was produced by Sigma Co. All reagents were analytical grade, and the water used was double-distilled.

The samples were characterized by a D/max 2400 type X-ray powder diffractometer (Rigaku, JP), Zetasizer 300 HS nanoparticle size Zeta potential analyzer (Malvern, UK), TU-1900 double-beam UV spectrophotometer (Beijing Purkinje General Instrument Co.), and Philips XL-30 scanning electron microscope.

### 2.2. Preparation and Characterization of Submicron COM and COD

Preparation of COM crystallites was done as follows: up to 50 mL of 0.30 mol/L KAc and 50 mL of 0.30 mol/L CaCl_2_ were added into a 250 mL beaker at 30°C under intense stirring. Then, 50 mL of 0.30 mol/L K_2_C_2_O_4_ was rapidly added to the mixed solution. After reacting for 6 min, the suspension was centrifuged for 2 min at 4000 rpm. Finally, COM crystallites were collected and thoroughly washed with double-distilled water and ethanol alternately, and then vacuum dried at room temperature.

Preparation of COD crystallites was done as follows: after 50 mL of 0.30 mol/L sodium ammonia triacetate (Na-NTA) and 50 mL of 0.30 mol/L CaCl_2_ solution were mixed and intensely stirred for 30 min at 30°C, 50 mL of 0.30 mol/L K_2_C_2_O_4_ was added. After reacting for 5 min, the suspension was centrifuged at 4000 rpm for 2 min. Finally, COD crystallites were collected and thoroughly washed with double-distilled water and ethanol alternately and then vacuum dried at room temperature.

The purity, size, and morphology of the COM and COD crystallites were characterized by an X-ray diffractometer, nanoparticle size analyzer, and scanning electron microscope.

### 2.3. Effect of C_6_S on Aggregation, Zeta Potential, and Phase Change of COM and COD Crystallites

COM or COD crystallites were added to solutions of *c*(C_6_S) = 0, 0.05, 0.1, 0.2, 0.3, 0.5, 2, 5, and 8 mg/L and ultrasonically dispersed for 5 min. Up to 1.6 mmol/L of COM or COD suspension was formed. After the suspensions were stored at 37°C for 0 and 4 h (*t* = 0, 4 h), the optical densities of the suspensions were measured by a UV-vis spectrometer at 620 nm [24]. At *t* = 0, 24, 48, and 72 h, the particle sizes and Zeta potential of the crystallites were detected by a nanoparticle size Zeta potential analyzer, and the crystal components were measured by XRD. The mass percentage of COM and COD in the crystallites was calculated according to the literature [25].

### 2.4. Effect of C_6_S on Deposition of COM and COD Crystallites

After 2.4 mg COM and 2.6 mg COD were added to 12 mL solutions of *c*(C_6_S) = 0, 0.1, 0.5, and 5 mg/L, respectively, suspensions of COM and COD crystallites were formed. After ultrasonic dispersion for 5 min, the photos of the suspensions were taken at *t* = 0, 1, 2, 4, and 24 h. The effect of C_6_S concentration on the deposition of COM and COD crystallites was compared [26].

## 3. Results and Discussion

### 3.1. Characterization of Submicron COM and COD Crystallites

**Figure 1:**
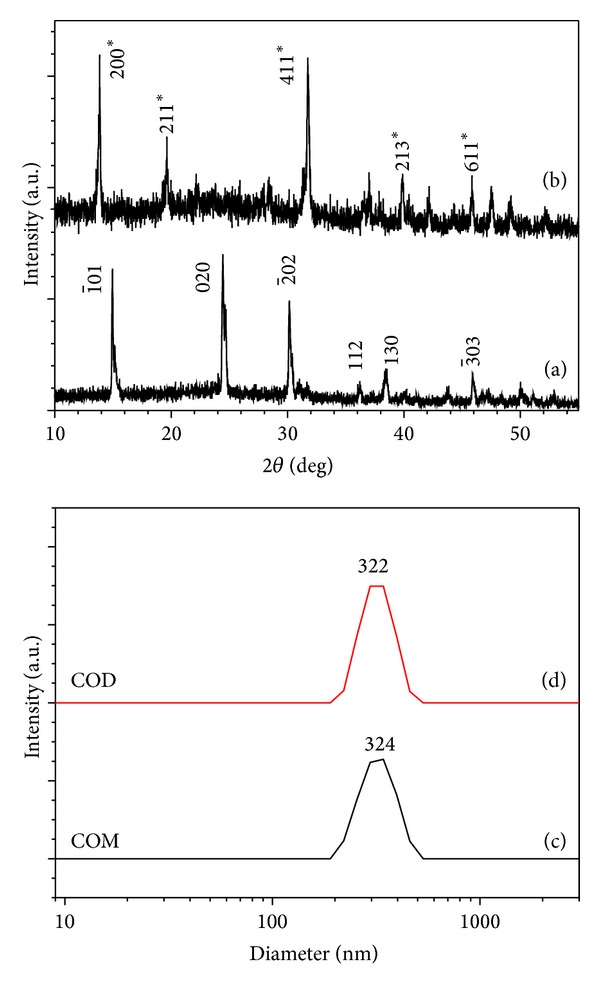
XRD patterns (a, b) and size distribution of submicron CaOxa (c, d). (a, c) COM; (b, d) COD.

**Figure 2:**
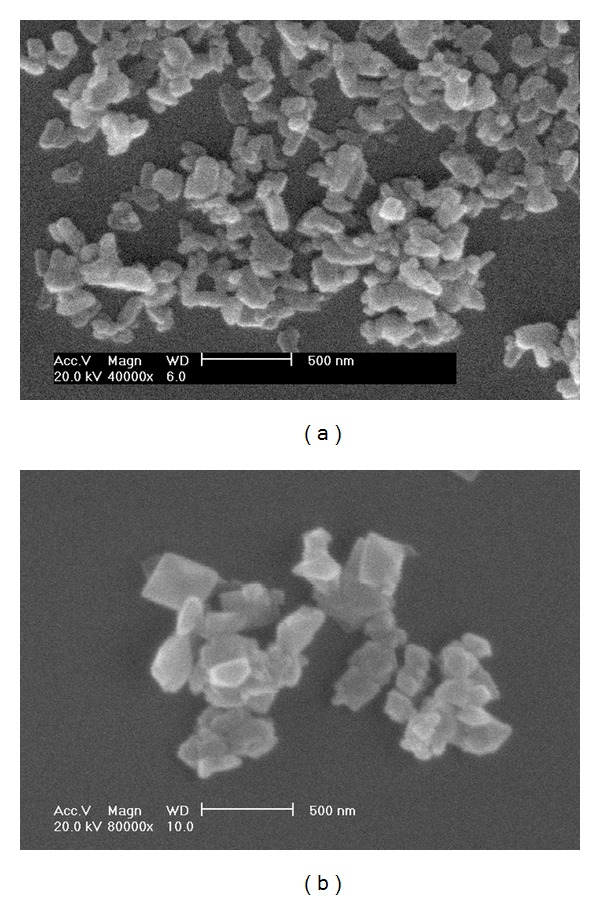
SEM images of submicron CaOxa. (a) COM; (b) COD.

The purity, particle size, and morphology of the products were identified by an X-ray powder diffractometer, nanoparticle size analyzer, and scanning electron microscope. The results shown in Figures 1 and 2 indicate that the samples were the target products of COM and COD crystallites, with mean particle sizes of about 330 nm.

### 3.2. C_6_S Inhibits COD Crystallites from Transforming into COM

**Figure 3:**
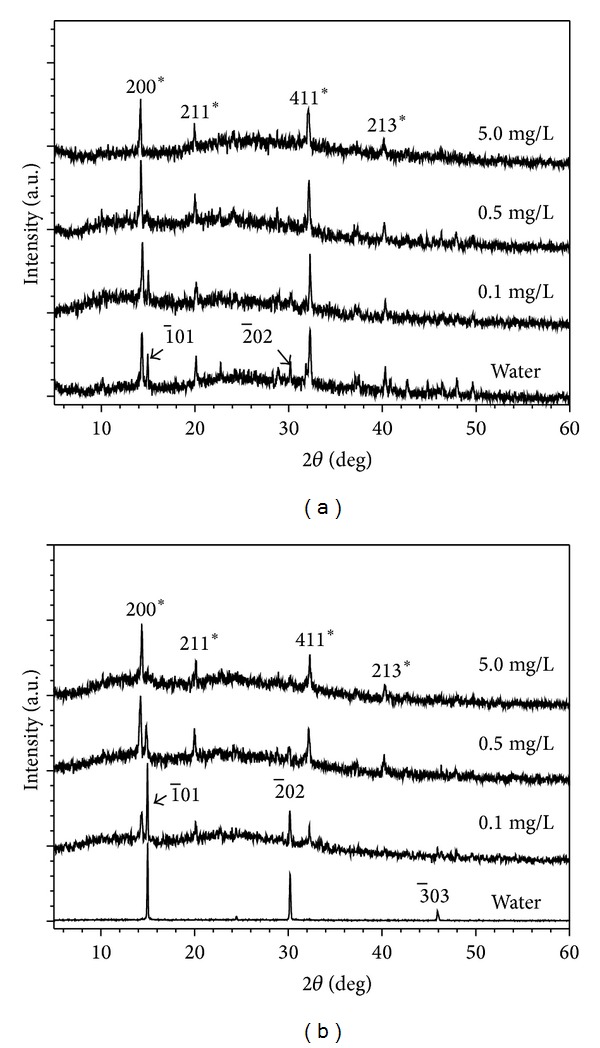
XRD patterns showing the effects of C_6_S concentration on conformation percentage of submicron COD into COM in the presence of different C_6_S concentrations and different acting times (*t*). (a) *t* = 48 h; (b) *t* = 72 h.

**Figure 4:**
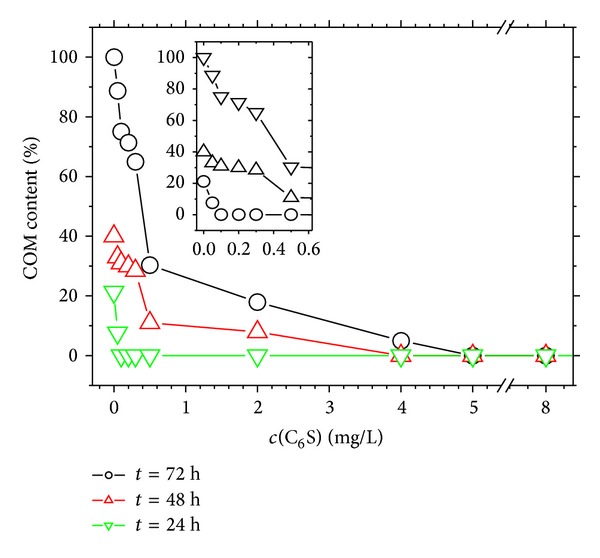
Effect of C_6_S concentration (*c*(C_6_S)) and acting time (*t*) on transformation of submicron COD to COM.

COM and COD crystallites are often aggregated in aqueous solution, whereas COD crystallites easily transform into COM crystallites because the latter is more stable in thermodynamics. The conformation percentage of submicron COD into COM in the presence of different C_6_S concentrations and different acting times (*t*) was detected by XRD. The detailed detection results of XRD patterns are shown in Figure 3 and the percentage of COM in the product calculated by the results of XRD is shown in Figure 4. The following premises were deduced from Figure 4.

1. At *c*(C_6_S) = 0, no C_6_S was found in the aqueous solution. The conversion percentage of COD was 21.3%, 37.7%, and 100%, respectively, at *t* = 24, 48, and 72 h.
2. At the same acting time, the conversion percentage of COD decreased with the increase in *c*(C_6_S). When *c*(C_6_S) ≥ 5.0 mg/L, no COD was converted to COM even at *t* = 72 h. This finding indicated that C_6_S could stabilize COD in aqueous solution.
3. COD slowly transformed to COM with the increase in *t*. The minimum C_6_S concentrations required to inhibit the conversion of submicron COD to COM in aqueous solution were *c*(C_6_S) = 0.1, 4, and 5 mg/L at *t* = 24, 48, and 72 h, respectively.
4. The stability of submicron COD in aqueous solution was less than that of micron-grade COD. A previous study [27] reported that a COD crystal with a size of 2 *μ*m to 3 *μ*m did not transform to COM crystal in water for 7 d until a small amount of COM seed was added. By contrast, submicron COD with a mean diameter of 330 nm could reach a higher conversion percentage within a short time in water solution. Thus, the smaller the size of the COD crystalis, the easier it can be converted to COM. COM crystallite is more harmful to the renal epithelial cell than COD, and its adhesive capacity on the cell is stronger than that of COD [28], so the inhibition of C_6_S of the transformation of COD to COM is helpful for preventing the formation of CaOxa stones.

### 3.3. C_6_S Induces the Zeta Potential on the Surface of COM and COD Crystallites to Become Negative

**Figure 5:**
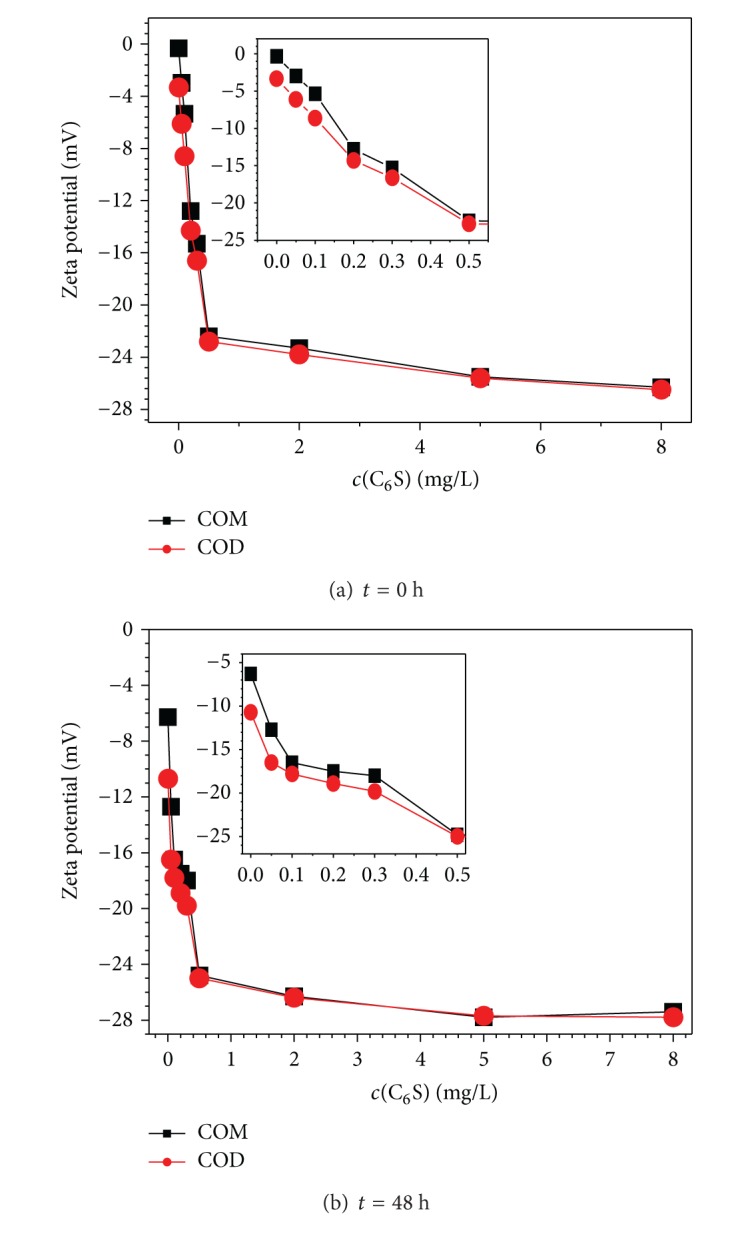
Effect of C_6_S concentration and acting time on Zeta potential of submicron COM and COD crystallites. (a) *t* = 0; (b) *t* = 48 h. The insets show the amplification figures when *c*(C_6_S) ≤ 0.5 mg/L.

The Zeta potential can reflect the charge on a particle surface. The surface Zeta potential of COM and COD crystallites in the presence of different *c*(C_6_S) concentrations is shown in Figure 5. The following were the findings obtained.

1. The presence of C_6_S induced the surface Zeta potential of both COM and COD crystallites to become negative, which was attributed to the absorbance with negatively charged C_6_S on the surface of the crystallites. With the increase of *c*(C_6_S), more C_6_S was absorbed on the surface of the crystallites, so their Zeta potential became more negative. After the Zeta potential became highly negative, the electrostatic repulsion force between the crystallites increased. Therefore, the aggregation and deposition of the crystallites were inhibited, which was beneficial in restraining the formation of CaOxa stones.
2. When *c*(C_6_S) ≤ 0.5 mg/L, the Zeta potential of the crystallites quickly decreased with the increase in *c*(C_6_S). When *c*(C_6_S) ≥ 0.5 mg/L, their Zeta potential value slowly became negative, indicating that the adsorption of C_6_S on the crystallite surface reached saturation at *c*(C_6_S) = 0.5 mg/L.
3. Comparing the adsorption capability of COM and COD by C_6_S molecules, the Zeta potential of COD was slightly more negative than that of COM when *c*(C_6_S) ≤ 0.5 mg/L, indicating that C_6_S was easier to adsorb on the surface of COD crystallites. This result was contrary to that of micron-grade COM and COD crystals because the main crystal face (101) of the hexagonal micron COM was positively charged and very large, whereas the sites with high charge densities of the octahedral bipyramidal micron COD were only found at the apex of two vertexes of the crystal. Thus, the positive charges on micron COD were less than those on micron COM. However, when the size of COD decreased, the number of COD crystallites increased for the same mass of crystallites. The number of their pyramidal vertexes increased, so submicron COD had more charges than micron COD, and its adsorption to C_6_S was strengthened. By contrast, when the size of COM decreased, its main crystal face, the positively charged (101) face, became smaller. Therefore, the positive charges on the COM surface reduced, so the adsorption capability of submicron COM to C_6_S decreased. As a result, the surface Zeta potential of submicron COD became more negative. The density of calcium ions on the COD surface was not significantly different from that of COM [29]. One calcium ion exists in about 0.305 nm^2^ to 0.459 nm^2^ of surface COD and about 0.383 nm^2^ to 0.455 nm^2^ of surface COM. A previous study [30] reported that negatively charged osteopontin is more strongly adsorbed on COD surface than on COM surface.
4. Compared with *t* = 0 h, the Zeta potential of COM and COD crystallites became slightly negative at *t* = 48 h because the adsorbed C_6_S on the crystallite surface became tighter and more orderly with the increase of acting time. Thus, the absorbed amount of C_6_S molecules slightly increased.

### 3.4. C_6_S Inhibits the Aggregation of COM and COD Crystallites

**Figure 6:**
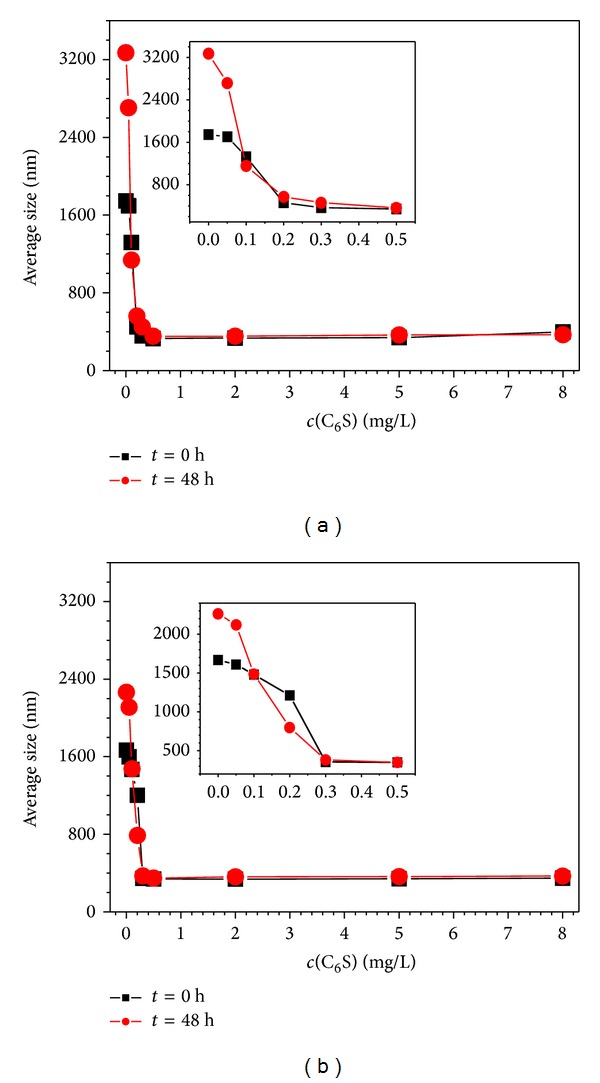
Effect of C_6_S concentration on the average size of submicron COM and COD. (a) COM; (b) COD. Acting time: 0 and 48 h.

**Figure 7:**
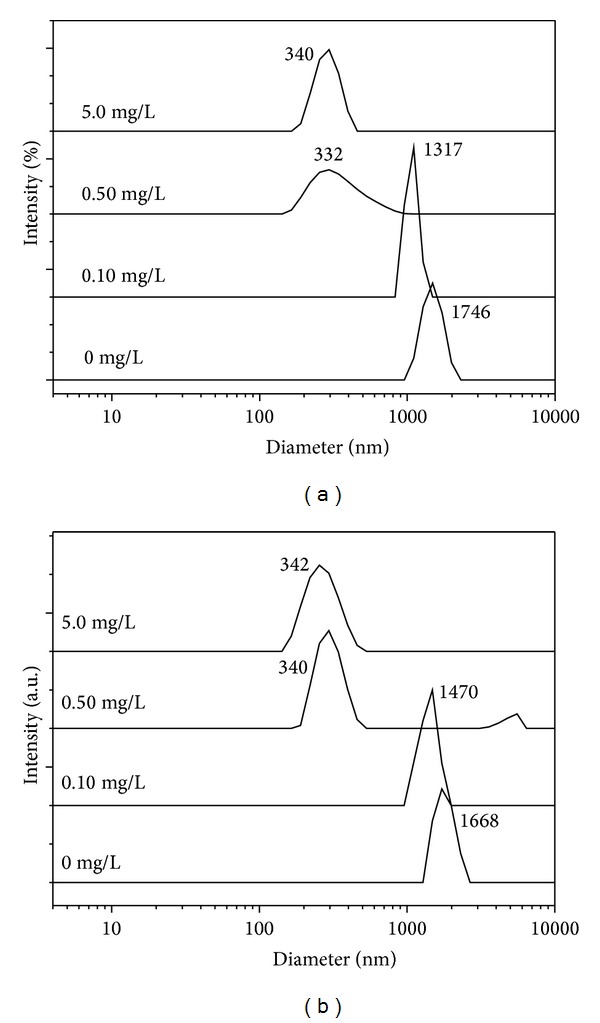
Effect of C_6_S concentration on size distribution of submicron COM and COD crystallites. (a) COM; (b) COD. *t* = 0 h.

A nanoparticle size analyzer was used to study the effect of C_6_S concentration on the mean diameters (Figure 6) and size distributions (Figure 7) of COM and COD particles. The results indicate the following.

**Figure 8:**
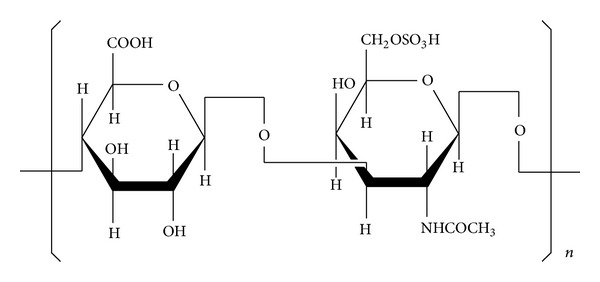
Chemical structure of chondroitin sulfate C (C_6_S).

1. At *c*(C_6_S) = 0, the crystallites were dispersed in pure water. The mean diameters of COM and COD were 1746 and 1668 nm, respectively, which were much larger than the initial mean diameter of about 330 nm (Figure 1(b)). This result indicates that the aggregation of both COM and COD crystallites in pure water was intensive.
2. When *c*(C_6_S) increased to 0.20 mg/L, the mean diameters of COM (Figure 6(a)) and COD (Figure 6(b)) were close to their initial diameters, which indicated that this concentration of C_6_S could entirely inhibit the aggregation of COM and COD in aqueous solution, inducing them to exist in a single crystal.
3. When *c*(C_6_S) increased from 0.5 mg/L to 2.0 mg/L, the size changes in COM and COD crystallites were small, and both were close to the particle size of a single crystal. If submicron COM and COD crystallites are considered spheres, we could calculate that every 2.4 and 3.0 nm^2^ surface area of COM and COD, respectively, adsorbed one unit of C_6_S molecule (one hexuronic acid and hexosamine unit, Figure 8) at *c*(C_6_S) = 0.5 mg/L. Due to limited sites of calcium ions on the crystallite surface and the effect of steric hindrance of C_6_S, the adsorption of C_6_S molecule on COM and COD crystallites was nearly saturated [31] at this point. The density of negative charges on the crystallite surface was the largest; that is, the Zeta potential on the crystallite surfaces became highly negative and the electrostatic repulsion between the crystallites was the strongest, so crystallite aggregation was inhibited [32].

When *c*(C_6_S) > 2.0 mg/L due to the excess adsorbed C_6_S around the crystallite in the solution, the crystallite size slightly increased.

### 3.5. C_6_S Inhibits Deposition of COM and COD Crystallites

**Figure 9:**
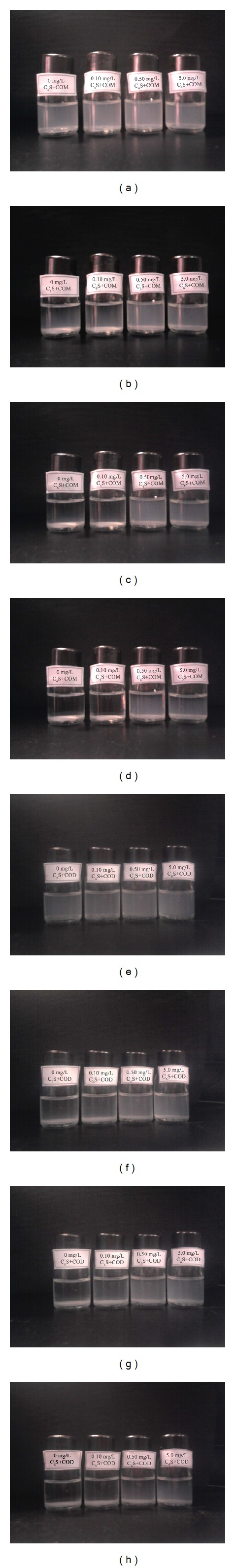
Photos showing sedimentation of the suspension of submicron COM and COD crystallites with placement time in presence of 0, 0.1, 0.5, and 5 mg/L of C_6_S, respectively. (a)–(d) COM; (e)–(f) COD. (a) and (e) 0 h; (b) and (f) 2 h; (c) and (g) 4 h; (d) and (h) 24 h.

Model tests *in vitro* and animal experiments confirmed that the retention of urine crystallites in renal tubules is one of the important factors of stone formation [33–35]. After COM and COD crystallites were dispersed in aqueous solutions of *c*(C_6_S) = 0, 0.1, 0.5, and 5 mg/L, photos of these suspensions at different placement times were taken (Figure 9), and their sedimentation speeds were compared.

1. At *t* = 0 h, all the crystallites were dispersed, regardless if they were COM or COD and whether the concentration of *c*(C_6_S) was 0 or 5.0 mg/L.
2. At *t* = 2 h, the suspensions of COM and COD crystallites in the presence of *c*(C_6_S) = 0 and 0.1 mg/L first sedimented and then their turbidities decreased. Submicron COM and COD had large specific surface areas and higher surface free energy, so they were easily aggregated and deposited in the aqueous solution when C_6_S was absent or when *c*(C_6_S) was very small.
3. At *t* = 4 h, the suspensions of crystallites in solutions of *c*(C_6_S) = 0 and 0.1 mg/L almost completely deposited. However, the turbidity of the suspensions of *c*(C_6_S) = 0.5 and 5.0 mg/L did not significantly decrease.
4. At *t* = 24 h, the turbidity of the suspensions of *c*(C_6_S) = 0.50 and 5.0 mg/L began to decline.

**Figure 10:**
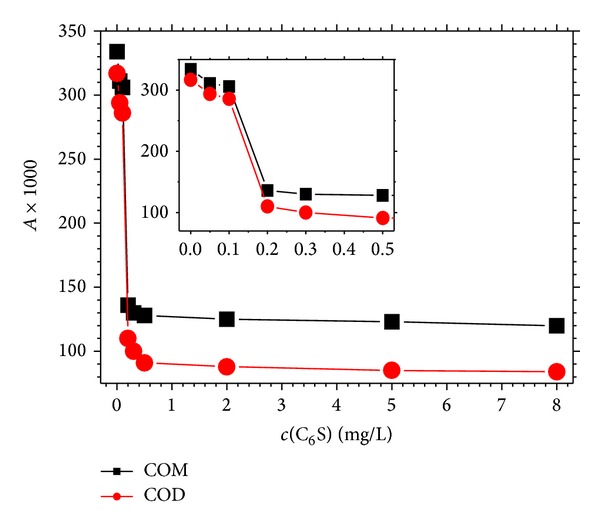
Sedimentation coefficient of suspensions of submicron COM and COD crystallites in the presence of different concentrations of C_6_S after placement for 4 h.

To quantify the sedimentation degree of COM and COD crystallites, a UV-vis spectrophotometer was used to measure the optical density of COM and COD suspensions after they were left to stand for 4 h (A_t=4_) at a wavelength of 620 nm [24]. According to the calculation method of aggregation coefficient in the literature [36], the differences in the optical densities at *t* = 4 h (*A*_*t*=4_) and *t* = 0 h (*A*_*t*=0_) were defined as sedimentation coefficient (SC):

(1) $$\begin{array}{c} \text{SC}=\left(A_{t=0}\text{–}A_{t=4}\right)\times 1000, \end{array}$$

where the value 1000 was used to convert SC into an integer number from a decimal number. The larger the SC, the greater the deposition degree of crystallites in the suspension. The results are shown in Figure 10.

1. In the suspension *c*(C_6_S) ≤ 0.10 mg/L, the SC values of both COM and COD were large, indicating that both COM and COD crystallites are easy to deposit in pure water or in diluted C_6_S solution.
2. At *c*(C_6_S) = 0.20 mg/L, the SC values rapidly declined from 306 and 286 to 136, 110, respectively, for COM and COD crystallites.
3. When *c*(C_6_S) ≥ 0.50 mg/L, the SC values of COM and COD slightly changed, which was attributed to the adsorption of C_6_S on the surface of COM and COD crystallites that reached saturation. Thus, the settlement of crystallites was effectively suppressed.
4. At the same *c*(C_6_S), the SC value of COD crystallites was slightly smaller than that of COM crystallites, indicating that the COD suspension was more stable than the COM suspension. This result was attributed to the following reasons. First, the mean particle diameter of COM and COD crystallites was nearly the same, but the density of COD crystallites (2.02 g/mL) was smaller than that of COM crystallites (2.20 g/mL). Moreover, the light COD crystallites were difficult to deposit. Second, one more crystal of water was present in the COD molecule than in the COM molecule, so the former more easily formed a hydrogen bond with the sulfate groups of C_6_S. Thus, the solubility of COD-C_6_S complex was larger than that of COM-C_6_S complex. Third, the Zeta potential of COD was slightly more negative than that of COM (Figure 5). Therefore, the electrostatic repulsion force between COD crystallites was slightly larger than that between COM crystallites. This finding can explain why stones did not easily form in the urine of healthy controls: the presence of more COD crystallites prevented their formation. By contrast, more COM crystallites were present in the urine of stone patients.

### 3.6. Inhibitory Mechanisms of C_6_S

GAGs are well-known macromolecular inhibitors found in urine and kidney stones [37]. C_6_S is the major component of GAGs responsible for their inhibitory effect. The inhibitory mechanisms of C_6_S include the following [38].

1. C_6_S is a linear polysaccharide polyanion. Each of its units has a negatively charged carboxylic group (uronic acid) and a negatively charged sulfate group [15]. These anions possess a strong capability to coordinate with Ca^2+^ ions. When C_6_S binds with free Ca^2+^, 1 *μ*mol chondroitin sulfate disaccharide unit binds with 0.757 *μ*mol Ca^2+^, leading to increased concentration of soluble Ca^2+^ ions in urine, reduction in the saturation of CaOxa crystals, and inhibition of the nucleation and growth of CaOxa crystals [39].
2. C_6_S can increase the absolute value of the Zeta potential of CaOxa crystals by adsorbing on the surface crystals, resulting in the increase of electrostatic repulsion force between crystals and the inhibition of growth and aggregation of CaOxa crystals [20, 40].
3. After their adsorption on the surface of CaOxa crystals, C_6_S molecules with numerous negative charges close the sites for crystal growth and aggregation, leading to defects in crystal growth and prevention of the penetration of free crystal particles.
4. C_6_S can protect the mucosa of the urinary tract, thus preventing the adhesion of bacteria or other free particles on the surface of the urinary crystals.

The intake of drugs to prevent stone formation can increase urine GAG excretion, as manifested by the following examples. (1) The oral administration of pentosan polysulphate (SPP) can increase urinary GAG excretion; in fact, SPP is extensively used in the treatment of patients with renal calcium stone disease [41]. (2) After oral administration of *Sterculia lychnophora* Hance (a Chinese herb medicine), the urine GAG excretion in patients with renal calculi increased from 29.27 ± 6.63 mg/24 h before drug intake to 35.94 ± 7.29 mg/24 h (*P* < 0.05) [42]. (3) The urine GAG excretion in patients with renal calculi increased from 31.2 ± 6.5 mg/24 h before drug intake to 46.4 ± 4.5 mg/24 h after the oral administration of *WuIing Powder* (a mixture of Chinese herb medicines) [43]. A significant difference was observed before and after the oral administration of *WuIing Powder*. (4) After K_3_cit intake for 1 week, the GAG excretion from the urine of all 13 cases of CaOxa stone formers increased from 5.18 ± 0.82 mg/L to 11.81 ± 1.62 mg/L [33], whereas that of the control was 9.80 ± 1.83 mg/L. A significant difference was observed before and after K_3_cit intake (*P* < 0.05).

Based on the discussion above, we can increase the excretion of chondroitin sulfate (i.e., GAGs) in urine through the intake of drugs to inhibit the formation of CaOxa calculi.

## 4. Conclusions

C_6_S could increase the Zeta potential on the surface of submicron COM and COD crystallites with mean sizes of 330 nm as well as the electrostatic repulsion force between the crystallites. Thus, C_6_S could inhibit aggregation and deposition of submicron COM and COD. C_6_S could also inhibit the transformation of COD to COM. At the same *c*(C_6_S) concentration, the Zeta potential of COD crystallites was more negative than that of COM, and the sedimentation coefficient of COD was smaller, so its suspension was more stable than that of submicron COM. These results indicated that C_6_S could inhibit the formation of CaOxa stones. Therefore, we can increase the excretion of chondroitin sulfate in urine through the intake of drugs to inhibit the formation of CaOxa calculi.
